# The importance of cache domains in α_2_δ proteins and the basis for their gabapentinoid selectivity

**DOI:** 10.1080/19336950.2023.2167563

**Published:** 2023-02-03

**Authors:** Karen M Page, Vadim M Gumerov, Shehrazade Dahimene, Igor B Zhulin, Annette C Dolphin

**Affiliations:** a Department of Neuroscience, Physiology and Pharmacology, University College London, London, UK; b Department of Microbiology and Translational Data Analytics Institute, The Ohio State University, Columbus, OH, USA

**Keywords:** Voltage-gated calcium channel, gabapentinoid, cache domain, amino acid, α_2_δ protein

## Abstract

In this hybrid review, we have first collected and reviewed available information on the structure and function of the enigmatic cache domains in α_2_δ proteins. These are organized into two double cache (dCache_1) domains, and they are present in all α_2_δ proteins. We have also included new data on the key function of these domains with respect to amino acid and gabapentinoid binding to the universal amino acid–binding pocket, which is present in α_2_δ-1 and α_2_δ-2. We have now identified the reason why α_2_δ-3 and α_2_δ-4 do not bind gabapentinoid drugs or amino acids with bulky side chains. In relation to this, we have determined that the bulky amino acids Tryptophan and Phenylalanine prevent gabapentin from inhibiting cell surface trafficking of α_2_δ-1. Together, these novel data shed further light on the importance of the cache domains in α_2_δ proteins.

## Introduction

The identification of a human variant in a Cache domain within α_2_δ-1 that contributes to a phenotype severely affecting neural development and function [1] has prompted this review of α_2_δ structure and function, in order to further understand the function of the cache domains in these multi-domain proteins. In addition, we present further experimental data related to the specificity and importance of amino acid and gabapentinoid binding to the amino acid–binding site in the first double cache domain.

## Classical role of α_2_δ in a complex within calcium channels

Voltage-gated calcium channels were first purified and the genes cloned from skeletal muscle in the 1980s [2,3]. The α_2_δ subunit was identified as one of the subunits, which was associated with the dihydropyridine receptor (α1 subunit) that was identified as a pore-forming subunit of the skeletal muscle calcium channel. Once the α_2_δ subunits were purified and cloned [4,5], they were also found to associate with N-type and P/Q-type channels, as well as other L-type channels [6,7]. The α_2_δ subunits are now known to associate with and affect the function of all Ca_V_1 and Ca_V_2 channels [8–11].

## α_2_δ subtypes

The skeletal muscle α_2_δ protein, termed α_2_δ-1, is encoded by *CACNA2D1*, which was the first α_2_δ gene to be cloned [12,13]. Four α_2_δ subunit genes were eventually cloned: *CACNA2D2*, encoding α_2_δ-2, was identified as a result of finding spontaneous mouse mutations leading to cerebellar ataxia and absence epilepsy [14,15]. *CACNA2D3* and *CACNA2D4* encoding α_2_δ-3 [11] and α_2_δ-4 [16], respectively, were then identified by homology to the α_2_δ-1 sequence.

## α_2_δ distribution and functions

The skeletal muscle α_2_δ protein, α_2_δ-1, was found also to be present extensively in other mainly excitable cell types, including those in the heart, smooth muscle, and brain [17]. In neurons, it is particularly concentrated presynaptically, and it is involved in presynaptic functions including transmitter release, homeostatic plasticity, and synaptic organization [18–21]. In contrast, the tissue distribution of α_2_δ-2 was found to be mainly in the brain, particularly the cerebellum, but also in other tissues [11,14], and α_2_δ-3 was expressed widely in the brain, particularly in the caudate-putamen [11]. The selective distribution and importance of α_2_δ-4 in retinal function was elucidated by virtue of its mutation in hereditary retinal dysfunction [22,23].

## Biochemistry and domains within α_2_δ

**Figure 1.**
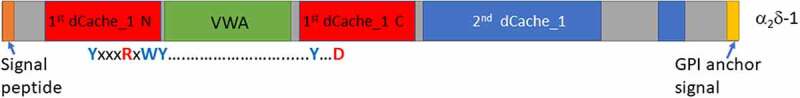
Domain structure of α_2_δ-1 The amino acid–binding site motif in the first dCache_1 domain is shown beneath the linear domain representation .[35]. The R and D drawn in red in the motif have been mutated in binding studies described here. Modified from Figure 3c in [35].

All α_2_δ proteins have similar topology, biochemical properties, and domain architecture (Figure 1). Both α_2_ and δ are highly N-glycosylated with up to 16 glycosylation sites [13,24,25], in agreement with their extracellular topology. All are proteolytically cleaved into two polypeptides, the larger α_2_, and the smaller δ [26]. These remain disulfide-bonded together [13]. The C-terminal hydrophobic domain is present in all α_2_δ pre-protein sequences [26]. Although this hydrophobic domain was originally predicted to be transmembrane [25], it was found to contain key glycosyl-phosphatidylinositol (GPI)-anchor signal motifs for all the α_2_δ sequences [27], which was confirmed in biochemical, functional, and structural studies [27,28]. Thus, the C-terminal hydrophobic domain and short putative intracellular sequence, translated in the α_2_δ pre-protein are removed by processing in the endoplasmic reticulum, being replaced by a lipid anchor, and are therefore absent from the mature α_2_δ protein present in the calcium channel complex [28,29].

There is also a von Willebrand factor-A (VWA) domain in α_2_, [30] which is a well-recognized protein–protein interaction domain, that is also present in many other extracellular proteins, including integrins [31]. The VWA domain in α_2_δ proteins is required for enhancement of calcium current function [9]. In α_2_δ-1 and α_2_δ-2 subunits, the VWA domains have a characteristic completely intact metal ion-dependent adhesion site (MIDAS) motif [30,32]. In other VWA domains, such as those in integrins, this MIDAS motif co-ordinates binding to another protein ligand, which occurs in the presence of a divalent cation, and which results in a conformational change [31]. In α_2_δ-1 and α_2_δ-2, disruption of the MIDAS motif prevents the ability of these α_2_δ subunits to enhance calcium channel currents [9,18]. The main corresponding interaction of the α_2_δ MIDAS motif with the Ca_V_ channels involves an aspartate in extracellular loop I of domain I of the α1 subunit, which coordinates with the α_2_δ MIDAS motif [28,33]. However, the structure also shows an additional interaction between a loop of the first Cache domain of α_2_δ-1 with the top of pore loop 5 in domain III, which forms part of the extracellular entrance to the channel pore [28].

## The importance of cache domains in α_2_δ proteins

The α_2_δ proteins were found to contain domains related to those in bacterial chemoreceptors that were termed Cache domains [34], and it was identified structurally that four Cache domains were present in α_2_δ-1 [28]. In α_2_δ proteins, as in some prokaryotic proteins, these were found to be organized into double Cache domains (dCache_1), and in bacteria, they are involved in amino acid nutrient binding in chemoreceptors and other signal transduction proteins, leading to intracellular signaling [35,36]. Although these domains are widely found in bacteria and archaea, where they have well-studied roles in nutrient sensing, the only animal proteins in which these dCache domains have been identified are α_2_δ proteins (Figure 1), and the novel α_2_δ-like protein Cachd1 [35], which is a transmembrane protein with some α_2_δ-like properties [33,37,38]

A conserved structural motif including several key residues was found to be essential for amino acid binding in all these dCache_1 domains, including in the first dCache_1 domain in α_2_δ-1 [35]. This dCache_1 domain is split in α_2_δ-1, with the VWA domain inserted into it. The presence of the VWA domain also splits the amino acid–binding motif. The motif (using the single letter amino acid code) consists of YxxxRxWY in the first cache domain and Y … D in the second cache domain (Figure 1). The Arg (R) in this motif (in red in Figure 1) was previously identified as being the third Arginine in the triple-Arg sequence that was found to be essential for gabapentin binding and for the function of gabapentinoids in alleviating neuropathic pain [39,40].

## Splicing creates variation in cache domains of α_2_δ proteins

**Figure 2.**
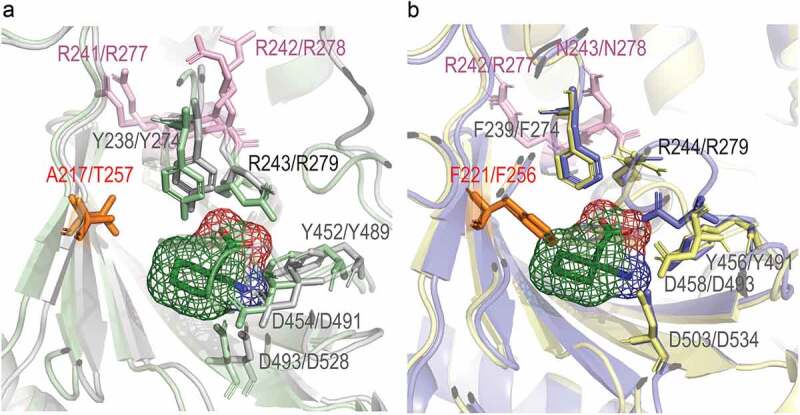
Gabapentinoid and amino acid–binding pockets of α_2_δ-1 – α_2_δ-4. Phe221/Phe256 (F221/F256) creates steric hindrance in the ligand-binding pocket of α_2_δ-3 and α_2_δ-4 proteins. Superimposed structures of α_2_δ-1 and α_2_δ-2 (a) and α_2_δ-3 and α_2_δ-4 (b) ligand-binding pockets. α_2_δ-1 is the rabbit protein cryo-EM structure, α_2_δ-2 to α_2_δ-4 are the AlphaFold models. Gabapentin is docked to the binding pocket of α_2_δ-1. Each of α_2_δ-2 to α_2_δ-4 was superimposed on α_2_δ1; α_2_δ-3 and α_2_δ-4 then were extracted and placed on panel B for clarity. A figure with all the proteins simultaneously superimposed on α_2_δ-1 can be found on GitHub at the following link https://github.com/ToshkaDev/Alpha2Delta-proteins-review. In pink font the first two residues of the RRR (in α_2_δ-1 and α_2_δ-2)/RNR (in α_2_δ-3 and α_2_δ-4) sequence are shown – as can be seen they are not involved in ligand binding. Other residues, except for the residue corresponding to Asp454 (D454) in α_2_δ-1, denote the amino acid–binding motif .[35]

Several different splice variants of the α_2_δ proteins have been identified [12,41,42]; these have been investigated most extensively in α_2_δ-1 and involve the cache domains. There are three regions of splicing in α_2_δ-1, termed A, B, and C; A and C are cassette exons, and B is introduced via an alternative splice acceptor site [43]. A and B are situated in the distal half of the first dCache_1 domain in a loop between β-sheet 6 and α-helix 7, whereas the third splice insertion, region C, is at the start of the second dCache_1 domain (see Figure 2 and Fig. S11, in [35]).

The three splice insertions in α_2_δ-1 are differentially expressed in different tissues [43,44]. These studies showed region A to be expressed exclusively in skeletal muscle from all the tissues examined. The rat skeletal muscle variant is +A + B ΔC, whereas in the rat brain the main splice variant is ΔA + B + C. A minor splice variant of α_2_δ-1 lacking region C (ΔA + B ΔC) is differentially up-regulated in rat dorsal root ganglion neurons following neuropathic injury, and it shows lower affinity for gabapentin [43]. The importance of the different splice insertions is unknown; it remains to be determined whether they are important for α_2_δ-1 structure and interaction with specific calcium channels such as, in the case of region A, the skeletal muscle channel α1S, or for interaction with other potential binding partners of α_2_δ-1 [45]. In this regard, it is of great interest that exogenous expression in hippocampal neurons of an α_2_δ-2 splice variant lacking exon 23, which is in an equivalent position to splice site C in α_2_δ-1 (see alignment in Fig. S11 in [35]), triggers aberrant synapse formation in tissue culture [46].

## Importance of α_2_δ proteins in disease in mouse and other animal models: Relevance to cache domains

Knockout mice have been generated for the different α_2_δ isoforms. From these studies, it is clear that the observed phenotype of particular α_2_δ knockout mice depends on the cell types and developmental stages associated with selective expression of the particular isoform, which may then become indispensable. The α_2_δ-1 knockout mice have a mild phenotype of reduced cardiac function, as α_2_δ-1 is strongly expressed in ventricular myocytes [47]. They also have a reduced sensation of mechanical pain [48], associated with the finding that α_2_δ-1 is strongly expressed in sensory neurons and is upregulated following neuropathic injury [49–51]. Furthermore, upregulated α_2_δ-1 mediates an increase in the trafficking of Ca_V_2.2 particularly in low threshold mechanoreceptors involved in hyperalgesia and allodynia [52]. Related to this, α_2_δ-1 knockout mice also exhibit delayed development of neuropathic pain-related responses [48]. Furthermore, transgenic mice that constitutively over-express α_2_δ-1 by random insertion [53] show spontaneous epileptiform behavior observed on EEG [54], and constitutive pain-like behavior [53]. In addition, auto-antibodies recognizing α_2_δ-1 are present in cases of autoimmune encephalitis [55] and amyotrophic lateral sclerosis with type 2 diabetes [56].

In contrast, α_2_δ-2 knockout mice [57] have a similar severe phenotype to the spontaneously arising *Ducky* and *entla* mutants, including cerebellar ataxia and epilepsy [14,15]. This phenotype relates to the fact that α_2_δ-2 is very strongly expressed in cerebellar Purkinje cells [14,58]. The phenotype of α_2_δ-3 knockout mice was more subtle, and included impaired acoustic startle response and hearing disruption [59].

*Drosophila melanogaster* has two α_2_δ orthologs, the skeletal muscle ortholog, *Ca-Ma2d*, and the α_2_δ-3 ortholog, *straightjacket* (*stg*) or dα_2_δ-3, which is important in neurotransmission [60]. Knockdown of *stg* gene expression results in impaired heat sensitivity [61]. Furthermore, single nucleotide polymorphisms (SNPs) in the human gene *CACNA2D3* have been associated with reduced behavioral noxious thermal sensitivity, likely via a central impairment [61].

Mutations in *Cacna2d4* result in disruption of retinal ribbon synapses in mice, as a result of both rod and cone dysfunction [23].

## Effect of human mutations in *CACNA2D* genes and relevance to cache domains

### Neurological disease

Several recent reviews cover the involvement of Ca_V_ channels in neurological and psychiatric disorders [62,63] and only a summary of recent studies relating to *CACNA2D* genes is provided here. In *CACNA2D2*, rare biallelic loss-of-function variation has been reported in individuals with developmental epileptic encephalopathy, including cerebellar atrophy [64–67]. Rare homozygous truncating mutations of *CACNA2D4* have been reported, which result in recessive, slowly progressing cone dystrophy and hereditary night blindness [22].

In *CACNA2D1*, biallelic loss-of-function mutations have also recently been reported in two patients with developmental epileptic encephalopathy, which is associated with cerebral cortical rather than cerebellar atrophy [1]. These individuals were also reported to be insensitive to pain. In one patient, there was a homozygous frameshift mutation, resulting in a marked reduction in *CACNA2D1* mRNA measured in the patient fibroblasts. The other patient was compound heterozygous for a very early frameshift mutation on one allele, and a point mutation (Gly209-Asp) on the other allele. This Gly209 was in a highly conserved residue in the first dCache_1 domain of α_2_δ-1 [1]. We found that this mutation rendered α_2_δ-1 nonfunctional, in that the mutant protein did not traffic to the cell surface. Our evidence further suggested that the mutant α_2_δ-1 was retained in the endoplasmic reticulum, since it was not proteolytically cleaved into α_2_ and δ, a process that occurs mainly in the Golgi apparatus [1,68].

## Genetic variation in *CACNA2D1*: Implications for cardiac disease in humans

In humans, heterozygous missense variations in *CACNA2D1* have previously been associated with cardiac dysfunction, with Brugada [69] and short QT [70] syndromes. However, these dominant associations with cardiac dysfunction have recently been called into question [1].

## Mechanism of action of gabapentinoid drugs and basis for their selectivity with respect to α_2_δ proteins

Gabapentin and pregabalin were first developed in drug discovery programs to identify novel antiepileptic drugs mimicking or promoting the function of the inhibitory neurotransmitter GABA [71]. These drugs were then identified to bind to α_2_δ-1 rather than their originally intended mechanism of action [72]. Mutational analysis then found the Arg mentioned above to be involved in gabapentinoid binding and function [39,40,73]. More recently, a key aspartate (Asp, D, Figure 1) was also identified as being essential to coordinate amino acid binding in this binding pocket, which is in the dCache_1 domain of α_2_δ-1 [35].

Regarding the mechanism of action of the gabapentinoids, we identified that gabapentin reduced the trafficking of α_2_δ-1 and α_2_δ-2 [74,75] and also disrupted the trafficking of associated calcium channels, and their function [75–78]. Within α_2_δ-1, both the key Arg241 [75,78] and also Asp491 [35] residues in the dCache_1 amino acid–binding site of α_2_δ-1 are important for the ability of gabapentin to inhibit α_2_δ-1 trafficking and function.

Of interest, α_2_δ-3 (and also α_2_δ-4) does not contain the triple Arg sequence that was thought to be implicated in gabapentin binding (it is Arg-Asn-Arg in α_2_δ-3), and neither α_2_δ-3 nor α_2_δ-4 binds gabapentin [79]. Furthermore, α_2_δ-3 is not recycled to the plasma membrane via a Rab11-dependent pathway [80].

Our analysis of the structures modeled by AlphaFold [81] shows that α_2_δ-3 and α_2_δ-4 do contain an amino acid–binding site, in an analogous position to that identified in the first dCache domain of α_2_δ-1 [35] (Figure 2). We conducted molecular docking in AutoDock Vina [82] with AlphaFold models of α_2_δ-2, α_2_δ-3 and α_2_δ-4 proteins using gabapentin, pregabalin, mirogabalin, and amino acids, and found that in case of α_2_δ-3 and α_2_δ-4, only small amino acids bind to the pocket, while gabapentinoids and bulky amino acids do not (structural models of α_2_δ-2, α_2_δ-3, and α_2_δ-4 proteins with docked ligands and docking simulation parameters can be found at this link: https://github.com/ToshkaDev/Alpha2Delta-proteins-review). Interestingly, all proteinogenic amino acids and gabapentinoids were bound to α_2_δ-2, but tryptophan (Trp) was found to bind only in a certain pose and with low affinity, which contrasts with its high affinity binding to α_2_δ-1. Our structural analysis shows that the first two Arg residues of the above-mentioned triple-Arg motif are directed away from the pocket and in fact do not directly contribute to the formation of the ligand-binding interface (Figure 2a). Only the third Arg in this sequence, which is part of the universal amino acid–binding motif, is directed toward the inside of the pocket and binds ligands (Figure 2b, [35]). Thus, the two first residues of the triple-Arg sequence do not play a role in ligand binding and, therefore, replacement of the second Arg to Asn in this motif observed in α_2_δ-3 and α_2_δ-4 is not the reason for their inability to bind gabapentinoids. Our subsequent examination allowed us to identify the “culprit” – Phenylalanine (Phe) at a specific position within the ligand-binding pocket of α_2_δ-3 and α_2_δ-4 that creates a steric hindrance interfering with the binding of bulky ligands (Figure 2b). In α_2_δ-1 and α_2_δ-2, alanine (Ala217) and threonine (Thr257), respectively, are located at this position (see Figure 2), and they do not impede ligand binding.

**Figure 3.**
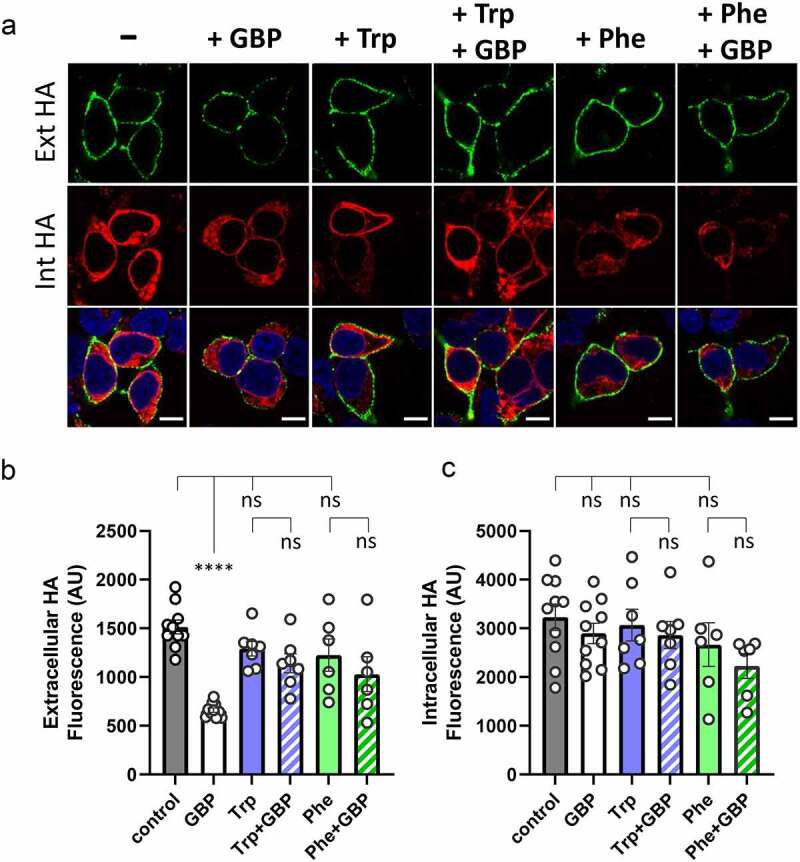
Tryptophan and Phenylalanine prevent the inhibition of cell surface expression of HA-tagged α_2_δ-1 by gabapentin. Experiments were performed as described previously described [35]. (a) Representative images of tsA-201 cells expressing hemagglutinin (HA)-tagged α_2_δ-1 subunit in the absence of gabapentin or additional amino acids (control, -) or the presence of 1 mM gabapentin (+ GBP) alone, 1 mM L-Tryptophan (+ Trp) alone, 1 mM L-Tryptophan + 1 mM gabapentin (+ Trp + GBP), 1 mM L-Phenylalanine (+ Phe) alone or 1 mM L-Phenylalanine + 1 mM gabapentin (+ Phe + GBP), incubated in serum-free media for 24 h. Top row (green, Ext HA) shows cell surface α_2_δ-1-HA staining in the nonpermeabilized condition; middle row (red, Int HA) shows intracellular α_2_δ-1-HA staining after permeabilization with 0.1% Triton X-100; bottom row shows merged images with the nuclei stained with DAPI (blue). Scale bars: 10 µm. (b) Bar chart (mean ± SEM, with individual data-points each showing the mean of more than 35 cells from 6–10 different transfections in three independent experiments), showing cell surface expression of α_2_δ-1-HA in the absence (control, gray) or presence of 1 mM GBP (white), 1 mM Trp (blue), 1 mM Trp + 1 mM GBP (blue and white stripes), 1 mM Phe (green), 1 mM Phe + 1 mM GBP (green and white stripes). Statistical significance was determined using one-way ANOVA and Šídák’s multiple comparison post-hoc test; **** P < 0.0001, ns: no statistical significance (P > 0.2). (c) As for (B) but showing intracellular HA staining after permeabilization of the cells. Cell surface expression of α_2_δ-1-HA is reduced by GBP to 44% of control levels but this reduction is not seen in the presence of additional L-Trp or L-Phe.

Bacterial chemoreceptors bind both agonists and antagonists at this universal amino acid–binding site within the dCache_1 domain [83,84]. For α_2_δ-1, the amino acid leucine was found previously to bind to the same binding site and compete with gabapentin, although the function of this binding was not known [85]. In our recent study, the binding affinity of various amino acids including Trp and Phe to α_2_δ-1 was calculated from docking analysis to be higher than that of leucine, and as high as that of the gabapentinoids [35].

We therefore examined here, using techniques already described [35], whether Trp or Phe would either inhibit α_2_δ-1 trafficking in the same way as gabapentin or, alternatively, act as agonists and enhance its trafficking. We found that although an elevated concentration (1 mM) of either Trp or Phe alone did not affect α_2_δ-1 cell surface expression in cultured cells, both these amino acids did inhibit the ability of gabapentin in this regard (Figure 3a-b). The cell surface expression of α_2_δ-1 was reduced by 56% by 1 mM gabapentin, as we have described previously [78,80], whereas this reduction was prevented by the additional presence of 1 mM Trp or Phe (Figure 3a, b). There were no effects of any of the manipulations on intracellular α_2_δ-1 expression (Figure 3a, c).

These results indicate that, although endogenous amino acids are likely to occupy the universal amino acid–binding site in α_2_δ-1, we were unable to detect any effect of the binding of high concentrations of Trp or Phe on cell surface expression of α_2_δ-1, indicating that under the conditions used here they did not act alone as either agonists or antagonists, although they are able to prevent the effect of gabapentin, presumably by occupying the binding site. This may represent one mechanism that contributes to the variable efficacy of gabapentinoid drugs.

## Conclusion

Within Metazoa, cache domains are only found in α_2_δ proteins and in Cachd1. In these proteins, the four cache domains are organized into two double Cache (dCache_1) domains, and contain a universal amino acid–binding pocket, which in α_2_δ-1 and α_2_δ-2 also accommodates gabapentinoid drugs. Here we have examined, from a structural point of view, why α_2_δ-3 and α_2_δ-4 do not bind gabapentinoids or amino acids with bulky side chains. Furthermore, we have determined that the bulky amino acids Trp and Phe prevent gabapentin from inhibiting cell surface expression of α_2_δ-1. Altogether, this illustrates the importance of the cache domains in α_2_δ proteins. It also highlights that novel interactions of these cache domains are likely to be found in the future.

## Data Availability

Structural data are available at https://github.com/ToshkaDev/Alpha2Delta-proteins-review. Other data will be made available upon reasonable request.
